# Special Report on Electrical Standards: New Internationally Adopted Reference Standards of Voltage and Resistance

**DOI:** 10.6028/jres.094.012

**Published:** 1989

**Authors:** B. N. Taylor

**Affiliations:** National Institute of Standards and Technology, Gaithersburg, MD 20899

**Keywords:** CCE, CIPM, Consultative Committee on Electricity, International Committee of Weights and Measures, International System of Units, Josephson effect, Josephson frequency-to-voltage quotient, ohm, quantum Hall effect, quantized Hall resistance, SI, volt

## Abstract

This report provides the background for and summarizes the main results of the 18th meeting of the Consultative Committee on Electricity (CCE) of the International Committee of Weights and Measures (CIPM) held in September 1988. Also included are the most important implications of these results. The principal recommendations originating from the meeting, which were subsequently adopted by the CIPM, establish new international reference standards of voltage and resistance based on the Josephson effect and the quantum Hall effect, respectively. The new standards, which are to come into effect starting January 1, 1990, will result in improved uniformity of electrical measurements worldwide and their consistency with the International System of Units or SI. To implement the CIPM recommendations in the U.S. requires that, on January 1, 1990, the value of the U.S. representation of the volt be increased by about 9.26 parts per million (ppm) and the value of the U.S. representation of the ohm be increased by about 1.69 ppm. The resulting increases in the U.S. representations of the ampere and watt will be about 7.57 ppm and 16.84 ppm, respectively. The CCE also recommended a particular method, affirmed by the CIPM, of reporting calibration results obtained with the new reference standards that is to be used by all national standards laboratories.

## 1. Background

The 18th meeting of the Consultative Committee on Electricity (CCE) of the International Committee of Weights and Measures (CIPM) was held September 27 and 28, 1988, at the International Bureau of Weights and Measures (BIPM), which is located in Sevres (a suburb of Paris), France. NIST Director E. Ambler, a member of the CIPM and President of the CCE, chaired the meeting and the author attended as NIST representative. Some 30 individuals from 15 countries participated.

As discussed in this journal in the author’s 1987 report on the 17th meeting of the CCE held at the BIPM in September 1986 [1], the CCE is one of eight CIPM Consultative Committees which together cover most of the areas of basic metrology. These Committees give advice to the CIPM on matters referred to them. They may, for example, form “Working Groups” to study special subjects and make specific proposals to the CIPM concerning changes in laboratory reference standards and in the definitions of units. As organizational entities of the Treaty of the Meter, one of the responsibilities of the Consultative Committees is to ensure the propagation and improvement of the International System of Units or SI, the unit system used throughout the world. The SI serves as a basis for the promotion of long-term, worldwide uniformity of measurements which is of considerable importance to science, commerce, and industry.

However, scientific, commercial, and industrial requirements for the long-term repeatability and worldwide consistency of voltage and resistance measurements often exceed the accuracy with which the SI units for such measurements, the volt^1^ and the ohm, can be readily realized. To meet these severe demands, it is necessary to establish representations^1^ of the volt and ohm that have a long-term reproducibility and constancy superior to the present direct realizations of the SI units themselves.

Indeed, as discussed by the author in reference [1], in 1972 the CCE suggested that the national standards laboratories adopt 483 594 GHz/V exactly as a conventional value of the Josephson frequency-to-voltage quotient for use in maintaining an accurate and reproducible representation of the volt by means of the Josephson effect. While most national laboratories did adopt this value, three decided to use different values. Moreover, it has become apparent that the CCE’s 1972 value of this quotient is about 8 parts per million (ppm) smaller than the SI value, implying that representations of the volt based on the 1972 value are actually about 8 ppm smaller than the volt.

It has also become apparent that because most national standards laboratories base their representation of the ohm on the mean resistance of a particular group of wire-wound resistors, the various national representations of the ohm differ significantly from each other and the ohm, and some are drifting excessively. Although the Thompson-Lampard calculable capacitor can be used to realize the ohm with an uncertainty^2^ of less than 0.1 ppm, it is a difficult experiment to perform routinely. Hence, the 1980 discovery of the quantum Hall effect (QHE) by K. von Klitzing [6] was enthusiastically welcomed by electrical metrologists because it promised to provide a method for basing a representation of the ohm on invariant fundamental constants in direct analogy with the Josephson effect. The QHE clearly had the potential of eliminating in a relatively straightforward way the problems of nonuniformity of national representations of the ohm, their variation in time, and their inconsistency with the SI.

To address the problems associated with current national representations of the volt and ohm as discussed above, the CCE at its 17th meeting established through Declaration E1 (1986),^3^ “Concerning the Josephson effect for maintaining the representation of the volt,” the CCE Working Group on the Josephson Effect. The CCE charged the Working Group to propose a new value of the Josephson frequency-to-voltage quotient consistent with the SI value based upon all relevant data that became available by June 15, 1988. Similarly, recognizing the rapid advances made in understanding the QHE since its comparatively recent discovery, the CCE established through Declaration E2 (1986),^3^ “Concerning the quantum Hall effect for maintaining a representation of the ohm,” the Working Group on the Quantum Hall Effect. The CCE charged the Working Group to (i) propose to the CCE, based upon all relevant data that became available by June 15, 1988, a value of the quantized Hall resistance consistent with the SI value for use in maintaining an accurate and stable national representation of the ohm by means of the QHE; and (ii) develop detailed guidelines for the proper use of the QHE to realize reliably such a representation.^4^

Further, the CCE stated its intention to hold its 18th meeting in September 1988 with a view to recommending that both the proposed new value of the Josephson frequency-to-voltage quotient and the proposed value of the quantized Hall resistance come into effect on January 1, 1990. These values would be used by all those national standards laboratories (and others) that base their representation of the volt on the Josephson effect, and that choose to base their representation of the ohm on the QHE. These proposals of the CCE were subsequently approved by the CIPM [8] and by the General Conference of Weights and Measures (CGPM) [9] under whose authority the CIPM functions.

In response to the CCE’s directives, each Working Group prepared a report which focused on the review and analysis of the values of the Josephson frequency-to-voltage quotient or quantized Hall resistance in SI units that were available by June 15, 1988; and the derivation of a recommended value for the purpose of establishing an accurate and internationally uniform representation of the volt and of the ohm based on the Josephson effect and on the quantum Hall effect, respectively. Submitted to the CCE in August 1988, the reports include useful background information as well as a discussion as to how the new representations might be used in practice to express calibration results. In keeping with the CCE’s charge, the QHE Working Group also prepared a companion report entitled “Technical Guidelines for the Reliable Measurement of the Quantized Hall Resistance.” Because unbiased quantized Hall resistance determinations are required for an accurate and reproducible representation of the ohm based on the QHE, these guidelines are of exceptional importance.^5^

## 2. CCE 18th Meeting Discussion and Principal Decisions

As an aid to the reader, this section of the report also includes some tutorial information.

### 2.1 Josephson Effect

#### 2.1.1 Definition of Josephson Constant

When a Josephson junction is irradiated with microwave radiation of frequency *f*, its current vs voltage curve exhibits steps at highly precise quantized Josephson voltages *U*_J_. The voltage of the *n*th step *U*_J_(*n*), *n* an integer, is related to the frequency of the radiation by

$$U_{J}(n)=nf/K_{J},$$ (1)

where *K*_J_ is commonly termed the Josephson frequency-to-voltage quotient [11]. The Working Group on the Josephson Effect (WGJE) proposed that this quotient be referred to as the Josephson constant and, since no symbol had yet been adopted for it, that it be denoted by *K*_J_. It follows from eq (1) that the Josephson constant is equal to the frequency-to-voltage quotient of the *n* = 1 step.

The theory of the Josephson effect predicts, and the experimentally observed universality of eq (1) is consistent with the prediction, that *K*_J_ is equal to the invariant quotient of fundamental constants 2*e*/*h*, where *e* is the elementary charge and *h* is the Planck constant [11]. For the purpose of including data from measurements of fundamental constants in the derivation of their recommended value of *K*_J_, the WGJE assumed that 2*e*/*h =K*_J_. However, *K*_J_ is not intended to represent the combination of fundamental constants 2*e*/*h.*

#### 2.1.2 Josephson Effect Reference Standard of Voltage

The CCE reviewed the report from the WGJE and discussed at some length the draft recommendation El (1988), “Representation of the volt by means of the Josephson effect,” prepared jointly by the WGJE and the Working Group on the Quantum Hall Effect. The CCE then agreed:

- to use the term “Josephson constant” with symbol *K*_J_ to denote the Josephson frequency-to-voltage quotient;
- to accept the WGJE’s recommended value of *K*_J_ namely, *K*_J_=(483 597.9±0.2) GHz/V, where the 0.2 GHz/V assigned one-standard-deviation uncertainty corresponds to a relative uncertainty of 0.4 ppm;
- to use this recommended value to define a conventional value of *K*_J_ and to denote it by the symbol *K*_J−90_ so that $K_{J-90}\overset{\text{def}}{=}483$ 597.9 GHz/V exactly. (The subscript 90 derives from the fact that this new conventional value of the Josephson constant is to come into effect starting January 1, 1990, a date reaffirmed by the CCE.) The CCE also noted
- that since *K*_J−90_ exceeds the CCE’s 1972 conventional value of the Josephson constant by 3.9 GHz/V or about 8.065 ppm, the new representation of the volt will exceed that based on the 1972 value by about 8.065 ppm; and further agreed
- that because the purpose of the new volt representation is to improve the worldwide uniformity of voltage measurements and their consistency with the SI, laboratories which do not base their national representation of the volt on the Josephson effect should, on January 1, 1990, adjust the value of their national volt representation so that it is consistent with the new representation. Further, this consistency should be maintained by having a transportable voltage standard periodically calibrated by a laboratory that does base its representation of the volt on the Josephson effect;
- that even if future, more accurate measurements of *K*_J_ indicate that the recommended value differs from the SI value by some small amount, the conventional value *K*_J−90_ should not be altered. Rather, the CCE could simply note the difference between a representation of the volt based on *K*_J−90_ and the volt; and
- that because an accurate representation of the volt is important to science, commerce, and industry, laboratories should continue their efforts to realize the volt with greater accuracy, either directly or indirectly via measurements of fundamental constants. This could lead to a significant reduction in the uncertainty assigned to the new volt representation.

Having concurred on these points, the CCE edited the draft recommendation E1 (1988) to bring it to final form. The following week it was submitted to the CIPM for approval at its 77th meeting held on October 4–6, 1988, at the BIPM. After some minor editorial changes, the CIPM adopted it as its own recommendation [12]. The following is the English language version (the French language version is the official one and is given in references [2] and [12]):

## Representation of the Volt by Means of the Josephson Effect Recommendation 1 (CI-1988)

The Comité International des Poids et Mesures, *acting* in accordance with instructions given in Resolution 6 of the 18th Conférence Générale des Poids et Mesures concerning the forthcoming adjustment of the representations of the volt and the ohm,

considering
  —that a detailed study of the results of the most recent determinations leads to a value of 483 597.9 GHz/V for the Josephson constant, *K*_J_, that is to say, for the quotient of frequency divided by the potential difference corresponding to the *n* = 1 step in the Josephson effect,
—that the Josephson effect together with this value of *K*_J_ can be used to establish a reference standard of electromotive force having a one-standard-deviation uncertainty with respect to the volt estimated to be 4 parts in 10^7^, and a reproducibility which is significantly better,
  recommends
—that 483 597.9 GHz/V exactly be adopted as a conventional value, denoted by *K*_J−90_, for the Josephson constant, *K*_J_,
—that this new value be used from 1st January 1990, and not before, to replace the values currently in use,
—that this new value be used from this same date by all laboratories which base their measurements of electromotive force on the Josephson effect, and
—that from this same date all other laboratories adjust the value of their laboratory reference standards to agree with the new adopted value,
  is of the opinion
—that no change in this recommended value of the Josephson constant will be necessary in the foreseeable future, and

*draws the attention* of laboratories to the fact that the new value is greater by 3.9 GHz/V, or about 8 parts in 10^6^ than the value given in 1972 by the Comité Consultatif d’Électricité in its Declaration E-72.

### 2.2 Quantum Hall Effect

#### 2.2.1 Definition of the von Klitzing Constant

The QHE is characteristic of certain high mobility semiconductor devices of standard Hall-bar geometry when in a large applied magnetic field and cooled to a temperature of about one kelvin. For a fixed current *I* through a QHE device there are regions in the curve of Hall voltage vs gate voltage, or of Hall voltage vs magnetic field depending upon the device, where the Hall voltage *U*_H_ remains constant as the gate voltage or magnetic field is varied. These regions of constant Hall voltage are termed Hall plateaus. Under the proper experimental conditions, the Hall resistance of the *i*th plateau *R*_H_(*i*) defined as the quotient of the Hall voltage of the *i*th plateau to the current *I*, is given by

$$R_{H}(i)=U_{H}(i)/I=R_{K}/i,$$ (2)

where *i* is an integer [13]. Because *R*_H_(*i*) is often referred to as the quantized Hall resistance regardless of plateau number, the Working Group on the Quantum Hall Effect (WGQHE) proposed that to avoid confusion, the symbol *R*_K_ be used as the Hall voltage-to-current quotient or resistance of the *i* = 1 plateau and that it be termed the von Klitzing constant after the discoverer of the QHE. It thus follows from eq (2) that *R*_K_=*R*_H_(1).

The theory of the QHE predicts, and the experimentally observed universaUty of eq (2) is consistent with the prediction, that *R*_K_ is equal to the invariant quotient of fundamental constants *h*/*e*^2^ [13]. For the purpose of including data from measurements of fundamental constants in the derivation of their recommended value of *R*_K_, the WGQHE assumed that *h*/*e*^2^ = *R*_K_. However, in analogy with *K*_J_, *R*_K_ is not intended to represent the combination of fundamental constants *h*/*e*^2^.

#### 2.2.2 Quantum Hall Effect Reference Standard of Resistance

The CCE reviewed the report of the WGQHE and discussed the draft recommendation E2 (1988), “Representation of the ohm by means of the quantum Hall effect,” prepared jointly by the two Working Groups. Because of the similarities between the QHE and the Josephson effect, the review and discussion proceeded expeditiously. Indeed, the second half of point (iii) as given here in section 2.1.2 on the Josephson effect and all of points (v), (vi), and (vii) were viewed by the CCE as applying to the quantum Hall effect as well. Also in analogy with the Josephson effect, the CCE agreed:

- to use the term “von Klitzing constant” with symbol *R*_K_ to denote the Hall voltage to current quotient or resistance of the *i* = 1 plateau;
- to accept the WGQHE’s recommended value of *R*_K_, namely, *R*_K_=(25 812.807±0.005) Ω, where the 0.005 Ω assigned one-standard-deviation uncertainty corresponds to a relative uncertainty of 0.2 ppm; and
- to use this recommended value to define a conventional value of *R*_K_ and to denote it by the symbol *R*_K−90_, so that $R_{K-90}\overset{\text{def}}{=}25$ 812.807 Ω exactly.

The same procedure was followed for draft recommendation E2 (1988) as for E1 (1988) regarding the Josephson effect. The final CIPM English language version is as follows:

## Representation of the Ohm by Means of the Quantum Hall Effect Recommendation 2 (CI-1988)

The Comité International des Poids et Mesures, *acting* in accordance with instructions given in Resolution 6 of the 18th Conférence Générale des Poids et Mesures concerning the forthcoming adjustment of the representations of the volt and the ohm,

Considering
  —that most existing laboratory reference standards of resistance change significantly with time,
—that a laboratory reference standard of resistance based on the quantum Hall effect would be stable and reproducible,
—that a detailed study of the results of the most recent determinations leads to a value of 25 812.807 Ω for the von Klitzing constant, *R*_K_, that is to say, for the quotient of the Hall potential difference divided by current corresponding to the plateau *i* = 1 in the quantum Hall effect,
—that the quantum Hall effect, together with this value of *R*_K_. can be used to establish a reference standard of resistance having a one-standard-deviation uncertainty with respect to the ohm estimated to be 2 parts in 10^7^, and a reproducibility which is significantly better,
  recommends
—that 25 812.807 Ω exactly be adopted as a conventional value, denoted by *R*_K−90_. for the von Klitzing constant, *R*_K_,
—that this value be used from 1st January 1990, and not before, by all laboratories which base their measurements of resistance on the quantum Hall effect,
—that from this same date all other laboratories adjust the value of their laboratory reference standards to agree with *R*_K−90_,
—that in the use of the quantum Hall effect to establish a laboratory reference standard of resistance, laboratories follow the most recent edition of the “Technical Guidelines for Reliable Measurements of the Quantized Hall Resistance” drawn up by the Comité Consultatif d’Électricité and published by the Bureau International des Poids et Mesures,
  and *is of the opinion*
—that no change in this recommended value of the von Klitzing constant will be necessary in the foreseeable future.

### 2.3 Practical Implementation of Recommendations

As implied by the discussion of section 1, the results of voltage and resistance measurements, expressed in terms of representations of the volt and ohm based on the Josephson and quantum Hall effects, respectively, will have a higher precision than the same measurement results expressed in terms of the volt and ohm themselves. Indeed, this is one of the principal reasons for establishing such representations.^6^ The question arises, however, as to how such measurement results should be reported in practice. The Working Groups recognized that the potential for significant confusion internationally could best be eliminated by having each national standards laboratory adopt the same approach. To this end, in their reports the Working Groups identified and considered the advantages and disadvantages of three different approaches to the reporting problem, two of which are both rigorous and correct [2]. In the first, new “practical units” “V_90_” and “Ω_90_” are defined; in the second, new, so-called “conventional physical quantities” for electromotive force (and electric potential difference) and resistance, “*E*_90_” and “*R*_90_,” are defined.

The CCE discussed at length the three approaches identified by the Working Groups and concluded that there was an alternative solution, similar to the Working Groups’ third approach, that is also rigorous but avoids

- defining new practical units of emf and resistance that are likely to differ from the volt and ohm by small amounts and which would be parallel to and thus in competition with the volt and ohm. (Defining such units automatically leads to practical electrical units for current, power, capacitance, etc., thereby giving the appearance that a complete new system of electrical units has been established outside of the SI.) The CCE’s alternative solution also avoids
- defining new conventional physical quantities for emf and resistance which are likely to differ from traditional or true emf and resistance by small amounts. (Defining such quantities automatically leads to conventional physical quantities for current, power, capacitance, etc.; and to the peculiar situation of, for example, the same standard cell having both a conventional emf and a true emf.) Further, the alternative solution avoids
- the use of subscripts or other distinguishing symbols of any sort on either unit symbols or quantity symbols. (With the elimination of such subscripts and symbols, for example, those denoting particular laboratories or dates, the national standards laboratories can avoid giving the impression to the users of their calibration services that there is more than one representation of the volt and of the ohm in general use, that there may be significant differences among national realizations of the new volt and ohm representations, and that either the national realizations or the new representations differ significantly from the SI.)

The CCE’s solution, which was affirmed by the CIPM at its 77th meeting [12] and which all national standards laboratories are requested to follow, is indicated in the following variation of the example given by the CCE [2] (the treatment of resistance measurements is strictly analogous):

The emf *E* of an unknown standard cell calibrated in terms of a representation of the volt based on the Josephson effect and the conventional value of the Josephson constant *K*_J−90_, may be rigorously expressed in terms of the (SI) volt V as (to be specific):

E=(1.018 123 45)V±ϵ, (3)

where *ϵ* represents the total uncertainty, in volts, and is composed of the following two components: Δ*E*, the combined uncertainty associated with the calibration itself and with the realization of the Josephson effect volt representation at the particular standards laboratory performing the calibration; and Δ*A*, the uncertainty with which the ratio *K*_J−90_/*K*_J_ is known (i.e., it is assumed that *K*_J−90_/*K*_J_=1*±*Δ*A*). According to Recommendation 1 (CI-1988), Δ*A* is 4 parts in 10^7^ or 0.4 ppm (assigned one standard deviation).

Since, by international agreement, Δ*A* is common to all laboratories, the two uncertainties Δ*E* and Δ*A* need not be formally combined to obtain the total uncertainty *ϵ* but may be separately indicated. Hence, the measured emf *E* may be expressed as

E=(1.018 123 45)V±ΔE (4)

for all practical purposes of precision electrical metrology and trade, with Δ*A* appearing separately on the calibration certificate when the precision of the calibration warrants it. If, for example, Δ*E*/*E* is significantly greater than 0.4 ppm, Δ*A* may be omitted with negligible effect.

An example of the wording that might be used on a NIST Report of Calibration for a standard cell enclosure for the case where Δ*A* may not be omitted and which is a variation of the wording given in an example developed by the CCE [2], is as follows:

## Sample Hypothetical NIST Calibration Report

This standard cell enclosure was received (date) under power at its normal operating temperature.

The values given in the table below are based on the results of daily measurements of the differences between the emfs of the cells in this standard and those of NIST working standards calibrated in terms of the Josephson effect using the new conventional value of the Josephson constant internationally adopted for use starting January 1, 1990 (see Note A). The measurements were made in the period from (date) to (date).

**Table:** 

Cell number	emf (volts, V)	Uncertainty (microvolts, *μ*V)
1	1.018 119 85	0.27
2	1.018 133 77	0.27
3	1.018 126 42	0.27
4	1.018 141 53	0.27

(Information relating to the measurements and their uncertainties to be given here.)

## Note A

The value of the Josephson constant used in this calibration, namely, *K*_J−90_=483 597.9 GHz/V exactly, is that adopted by international agreement for implementation starting on January 1, 1990, by all national standards laboratories that base their national representation of the volt (i.e., their national “practical unit” of voltage) on the Josephson effect. Since all such laboratories now use the same conventional value of the Josephson constant while prior to this date several different values were in use, the significant differences which previously existed among the values of some national representations of the volt no longer exist. Moreover, the national standards laboratories of those countries that do not use the Josephson effect for this purpose are requested to maintain their own national representation of the volt so as to be consistent with the above conventional value of the Josephson constant, for example, through periodic comparisons with a laboratory that does use the Josephson effect. An ideal representation of the volt based on the Josephson effect and *K*_J−90_ is expected to be consistent with the volt as defined in the International System of Units (SI) to within an assigned relative one-standard-deviation uncertainty of 0.4 ppm (0.41 *μ*V for an emf of 1.018 V). Because this uncertainty is the same for all national standards laboratories, it has not been formally included in the uncertainties given in the table. However, its existence must be taken into account when the utmost consistency between electrical and nonelectrical measurements of the same physical quantity is required.

### 2.4 Future Work on Electrical Units

The ideas agreed upon by the CCE as given in point (vii) in Sect. 2.1.2 on the Josephson effect, and which apply equally as well to the quantum Hall effect, led the CCE to adopt the following formal recommendation which was also approved by the CIPM at its 77th meeting [12].

## Realization of the Electrical SI Units Recommendation E3 (1988)

The Comité Consultatif d’Électricité

recognizing
  —the importance to science, commerce and industry of accuracy in electrical measurements,
—the fact that this accuracy depends on the accuracy of the reference standards of the electrical units,
—the very close ties that now exist between electrical metrology and fundamental physical constants,
—the possibility of obtaining more accurate reference standards of the electrical units either directly from the realizations of their definitions or indirectly from measurements of fundamental constants, and
—the continuing need to compare among themselves independent realizations of the units and independent measurements of fundamental constants to verify their accuracy,
  recommends
—that laboratories continue their work on the electrical units by undertaking direct realizations of these units and measurements of the fundamental constants, and
—that laboratories pursue the improvement of the means for the international comparison of national standards of electromotive force and electrical resistance.

## 3. Conclusion

The apparatus currently being used by the national standards laboratories is such that the total experimental uncertainty associated with a particular national representation of the volt based on the Josephson effect generally lies in the range 0.01 to 0.2 ppm. As a consequence, with the worldwide adoption starting January 1, 1990, of the new conventional value of the Josephson constant *K*_J−90_, all national representations of the volt should be equivalent to within a few tenths of a ppm. Similarly, the total experimental uncertainty associated with the measurement of quantized Hall resistances also generally lies in the range 0.01 to 0.2 ppm. Hence, with the worldwide adoption starting on January 1, 1990, of a new representation of the ohm based on the QHE and the conventional value of the von KIitzing constant *R*_K−90_. all national representations of the ohm should also be equivalent to within a few tenths of a ppm. Moreover, these new national volt and ohm representations should be consistent with the volt and the ohm to better than 0.5 ppm.

**Figure 1:**
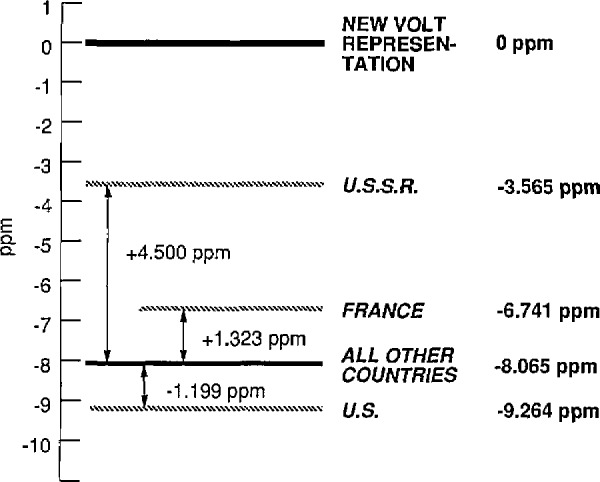
Graphical comparison of the value of the present representation of the volt of various countries as based on the Josephson effect, with the new representation of the volt based on the Josephson effect and the CIPM conventional value of the Josephson constant *K*_J−90_ which is to come into effect starting on January 1, 1990. The value of the volt representation indicated by “All Other Countries” is based on the conventional value of the Josephson constant stated by the CCE in 1972, namely, 483 594 GHz/V. The countries that currently use this value include Australia, Canada, Finland, F.R.G., G.D.R., Italy, Japan, The Netheriands, and the U.K. The BIPM uses this value as well, but NIST uses 483 593.420 GHz/V. Thus, as the figure shows, on January 1, 1990, the value of the present U.S. volt representation will need to be increased by 9.264 ppm to bring it into conformity with the new representation.

In the U.S., the value of the present national representation of the volt maintained by NIST will need to be increased on January 1, 1990, by about 9.26 ppm to bring it into agreement with the new representation of the volt. This is sufficiently large that literally thousands of electrical standards, measuring instruments, and electronic systems throughout the Nation will have to be adjusted or recalibrated in order to conform with the new representation. Most other countries will be required to make a similar change in the value of their present representation of the volt as can be seen from figure 1. On the same date, the value of the U.S. representation of the ohm maintained by NIST will need to be increased by about 1.69 ppm to bring it into agreement with the new representation of the ohm based on the quantum Hall effect. This too is an amount which is of significance to many existing standards, instruments, and systems. The change required in the value of the national representation of the ohm of other countries varies between a decrease of a few tenths of a ppm to an increase in excess of 3 ppm.

Since A=V/Ω where A is the ampere as defined in the SI; and W=V^2^/Ω where W is the watt as defined in the SI, the 9.264 ppm and 1.69 ppm increase in the U.S. representation of the volt and of the ohm, respectively, imply that on January 1, 1990, (i) the U.S. representation of the ampere will increase by about 7.57 ppm and (ii) the U.S. electrical representation of the watt will increase by about 16.84 ppm. Because an ideal volt representation based on the Josephson effect and *K*_J−90_ is expected to be consistent with the volt to within an assigned relative one-standard-deviation uncertainty of 0.4 ppm; and an ideal ohm representation based on the QHE and *R*_K−90_ is expected to be consistent with the ohm to within an assigned one-standard-deviation uncertainty of 0.2 ppm, ampere and watt representations derived from such ideal volt and ohm representations via the above equations are expected to be consistent with the ampere and watt to within a one-standard-deviation uncertainty of 0.45 ppm and 0.83 ppm, respectively.

The CCE strongly believes, and the author fully concurs, that the significant improvement in the international uniformity of electrical measurements and their consistency with the SI which will result from implementing the new representations of the volt and ohm will be of major benefit to science, commerce, and industry throughout the world; and that the costs associated with implementing the new representations will be far outweighed by these benefits.

## Footnotes

1 The volt is the SI unit of electromotive force (emf) and electric potential difference. Occasionally it may be referred to in the literature as the absolute volt. As-maintained volt, representation of the volt, laboratory representation of the volt, “national unit of voltage”, “laboratory unit of voltage”, “practical realization of the volt”, and other similar terms are commonly used to indicate a “practical unit” for expressing measurement results. However, to avoid possible misunderstanding, it is best not to use the word *unit* in this context. The only unit of emf in the SI is, of course, the volt. In keeping with references [2] and [3], from which this report has drawn heavily, we use the expression *representation of the volt* and variations thereof. The expression *reference standard of voltage* is also used occasionally in a similar or related sense. The situation for the ohm and resistance is strictly analogous.

2 Throughout, all uncertainties are meant to correspond to one standard deviation estimates in keeping with CIPM Recommendation 1 (CI-1986) [4,5].

3 The complete declaration is given in reference [1], but see also references [5] and [7].

4 The members of the CCE Working Group on the Josephson Effect were R. Kaarls, Van Swinden Laboratorimn (VSL), The Netherlands; B. P. Kibble, National Physical Laboratory (NPL), U.K.; B. N. Taylor, (NIST); and T. J. Witt, Coordinator (BIPM). The members of the CCE Working Group on the Quantum Hall Effect were F. Delahaye (BIPM); T. Endo, Electrptechnical Laborafpry (ETL), Japan; 0, C, Jones (NPL); V, Kose, Physikalisch-Technische Bundesanstalt (PTB), F. R. G.; B. N. Taylor, Coordinator (NIST); and B. M. Wood, National Research Council of Canada (NRCC), Canada.

5 The complete reports of the Josephson and Quantum Hall Effect Working Groups including the “Technical Guidelines” (Rapports BIPM 88/77, 88/8, and 88/9) will appear in the proceedings of the CCE’s 18th meeting [2]. Additionally, a combined, somewhat condensed version of the two reports may be found in reference [3] and the “Technical Guidelines” in reference [10].

6 As noted by the CCE [2], the Josephson and quantum Hall effects and the values *K*_J−90_ and *R*_k−90_ cannot be used to define the volt and ohm. To do so would require a change in the status of the permeability of vacuum *μ*_0_ from an exactly defined constant, thereby abrogating the definition of the ampere. It would also give rise to electrical units which would be incompatible with the definition of the kilogram and units derived from it.
